# Elevated doublecortin-like kinase 1 serum levels revert to baseline after therapy in early stage esophageal adenocarcinoma

**DOI:** 10.1186/s40364-019-0157-z

**Published:** 2019-03-08

**Authors:** Emily M. Christman, Parthasarathy Chandrakesan, Nathaniel Weygant, John T. Maple, William M. Tierney, Kenneth J. Vega, Courtney W. Houchen

**Affiliations:** 1 Department of Medicine, University of Oklahoma Health Sciences Center, Oklahoma City, OK USA; 2 Peggy and Charles Stephenson Cancer Center, Oklahoma City, OK USA; 3 Department of Medicine, Division of Gastroenterology and Hepatology, Augusta University-Medical College of Georgia, 1120 15th Street, AD 2226, Augusta, GA 30912 USA

**Keywords:** DCLK1, Doublecortin-like kinase 1, Esophageal adenocarcinoma, Cancer stem cell, ELISA

## Abstract

**Background:**

Barrett’s esophagus (BE) and esophageal adenocarcinoma (EAC) incidence has been increasing in the United States for greater than 30 years. For the majority of EAC patients, treatment is limited and prognosis poor. Doublecortin like kinase-1 (DCLK1) is a cancer stem cell marker with elevated expression in BE patients with high grade dysplasia and/or EAC. This prospective cohort study was designed to compare serum DCLK1 levels before and after EAC treatment with endoscopic mucosal resection (EMR) and/or radio-frequency ablation (RFA).

**Methods:**

Barrett’s esophagus patients with low or high-grade dysplasia (*n* = 9) and EAC patients (Stage I/II) eligible for treatment were enrolled (*n* = 14). Serum was obtained at enrollment and at end of treatment (EoT) where possible (*n* = 6). Normal control samples (*n* = 5) were obtained from patients with normal upper endoscopies. Serum was analyzed for DCLK1 protein content by ELISA. Kruskal-Wallis, Mann Whitney U, Pearson correlation, and Receiver Operating Characteristic tests were used to analyze the data.

**Results:**

Serum DCLK1 levels were increased by > 50% in Barrett’s Esophagus (*n* = 9) and EAC patients (*n* = 14) vs controls (n = 5, *p* = 0.0007). These levels were reduced > 50% at EoT compared to EAC (*p* = 0.033). Although age was significantly lower in controls, this factor was not statistically related to DCLK1 serum levels (*p* = 0.66).

**Conclusions:**

EAC treatment results in significantly decreased serum DCLK1 levels, suggesting that DCLK1 may be useful as a non-invasive disease regression biomarker following treatment.

**Impact:**

Biomarkers for EAC therapeutic response have been poorly studied and no reliable marker has been discovered thus far. These results demonstrate that DCLK1 may have potential as a circulating biomarker of the response to therapy in EAC, which could be used to improve patient outcomes.

## Background

According to population-based studies, it is estimated that at least 20% of Americans suffer from gastroesophageal reflux disease (GERD) [1]. This is a chronic condition related to the reflux of gastric contents into the esophagus which can result in inflammation of the squamous epithelium. Rarely, this condition leads to BE, which is the metaplastic transition of the normal esophageal epithelium from squamous to an intestinal-type, characterized by columnar epithelial cells [2]. BE is a known risk factor for the subsequent development of EAC. Evidence points to sequential progression from BE without dysplasia, to low grade dysplasia, then to high grade dysplasia (HGD), and ultimately EAC [3]. Unfortunately, the median survival of patients who develop EAC is only 15% at 5 years [4]. Furthermore, in the United States, the incidence of EAC has increased more than six-fold over the past 30 years [5].

Endoscopic mucosal resection (EMR) of early neoplastic lesions in BE has become increasingly important both diagnostically for esophageal cancer staging and therapeutically, as a definitive treatment method [6, 7]. Data suggest that radiofrequency ablation (RFA) of the residual BE after successful EMR of high-grade dysplasia or mucosal cancer can significantly reduce the rates of metachronous neoplasia or recurrence [8, 9]. However, this approach is associated with recurrence of BE in up to 17% of patients, suggesting incomplete eradication of the tumor stem cells [9]. Previous studies have shown that DCLK1 levels are elevated above baseline in serum and tissue of patients with BE with HGD and with EAC [10]. Further evaluation of the effect of treatment in BE and EAC on stem cell markers, such as DCLK1, will enhance understanding regarding recurrence or progression to cancer in these patients and stimulate continued improvement in treatment and survival. Therefore, the aim of the present investigation was to determine if serum DCLK1 levels vary before and after treatment with endoscopic mucosal resection (EMR) and/or radio-frequency ablation (RFA) or esophagectomy in patients with EAC.

## Patients/materials and methods

### Patients, procedures, and sample collection

Patients who presented to our medical center with a diagnosis of Barrett’s esophagus with low or high grade dysplasia or EAC (Stage I-II) were eligible for enrollment. Exclusion criteria were patients less than 18 years of age, pregnancy, contraindications to upper endoscopy or surgery, or history of gastrointestinal tract cancer or any other known or suspected cancers.

The protocol was approved by the University of Oklahoma Health Sciences Center institutional review board. 34 samples from patients with Barrett’s esophagus (*n* = 9), focal intra-mucosal EAC or Stage I-II EAC before (*n* = 14) or after treatment (*n* = 6), or normal upper endoscopy findings (controls; *n* = 5) were obtained from February to October 2015. Following informed consent, serum was collected at enrollment and during the patient’s end-of-treatment visit where possible.

### Enzyme-linked immunosorbent assay (ELISA)

Serum DCLK1 levels were determined using an ELISA kit commercially available from Cloud Clone Corporation Wuhan (Houston, TX) according to manufacturer instructions. Briefly, the 96-well plate coated with monoclonal antibody against DCLK1 was pre-blocked. Purified DCLK1 protein at different concentrations (0–10 ng/mL) was used to create a standard curve. Plasma samples were diluted 1:4 & 1:10 with phosphate-buffered saline (PBS). The diluted samples, along with the purified DCLK1 proteins, were then added to the pre-blocked 96-well plate and incubated for 2 h at room temperature. Next, the plate was incubated with biotinylated polyclonal antibody against DCLK1 for 1 h at room temperature. After 3 washes, the plate was incubated with Streptavidin conjugated with horseradish peroxidase (HRP) for 30 min at room temperature. Finally, the plate was developed with HRP substrate for 20 min and terminated using the kit stop solution. Absorbance (OD_450_) was measured using a microplate reader. The concentration of DCLK1 in the serum samples was determined using the fitted concentration/absorbance curve constructed from the standards.

### Statistical analysis

The Mann-Whitney U Test and the Kruskal-Wallis test were used for non-parametric comparisons between patient groups. Receiver-operating characteristic (ROC) analysis was used to estimate the sensitivity and specificity of serum DCLK1 for various comparisons. A *p*-value of less than 0.05 was considered statistically significant. All analyses were performed in Graphpad Prism 7.0.

## Results

A total of 34 patients were enrolled in this study. Of this number, 5 were control patients with normal findings at their endoscopy. The remaining 24 were individuals with focal intra-mucosal or stage I-II EAC undergoing treatment (*n* = 14) or at the end of treatment (EoT, *n* = 6, Table 1). Additionally, 9 patients presenting with low (*n* = 3) or high-grade (*n* = 6) Barrett’s esophagus were also included in the study. For treatment, 9 of the EAC patients underwent esophagectomy and the remainder, which were diagnosed with EAC limited to the mucosa, underwent EMR.

**Table 1:** Demographics

Disease State	14 EAC, 6 EoT, 9 BE, 5 controls (34 samples total)
Sex	8 females, 26 males
Ethnicity	33 Caucasian, 1 African-American
Median Age (range)	65 (35–83)

*EAC* esophageal adenocarcinoma, *EoT* end of treatment, *BE* Barrett’s esophagus

**Fig. 1:**
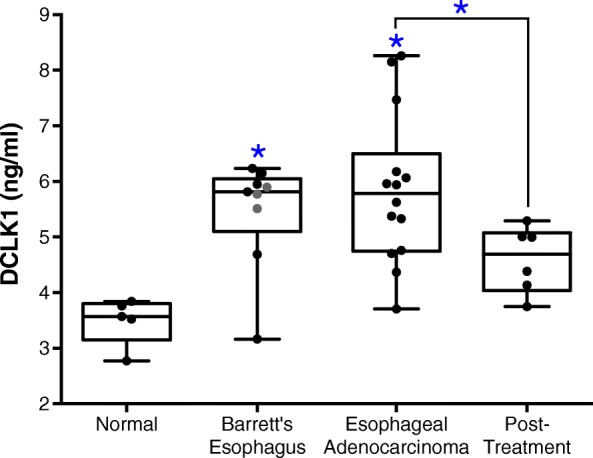
DCLK1 serum levels are significantly altered across normal (*n* = 5), Barrett’s esophagus (*n* = 9), and esophageal adenocarcinoma before (*n* = 14) and after treatment (*n* = 6)(*p* = 0.0029). Compared to normal controls, DCLK1 serum is increased 67.0% (95% CI: 45.0–89.7%) in stage I-II esophageal adenocarcinoma patients (*p* = 0.0007) and significantly decreased after treatment by approximately 50% (*p* = 0.0326). No statistically significant differences were apparent between low-grade (grey points) and high-grade (black points) Barrett’s esophagus

**Fig. 2:**
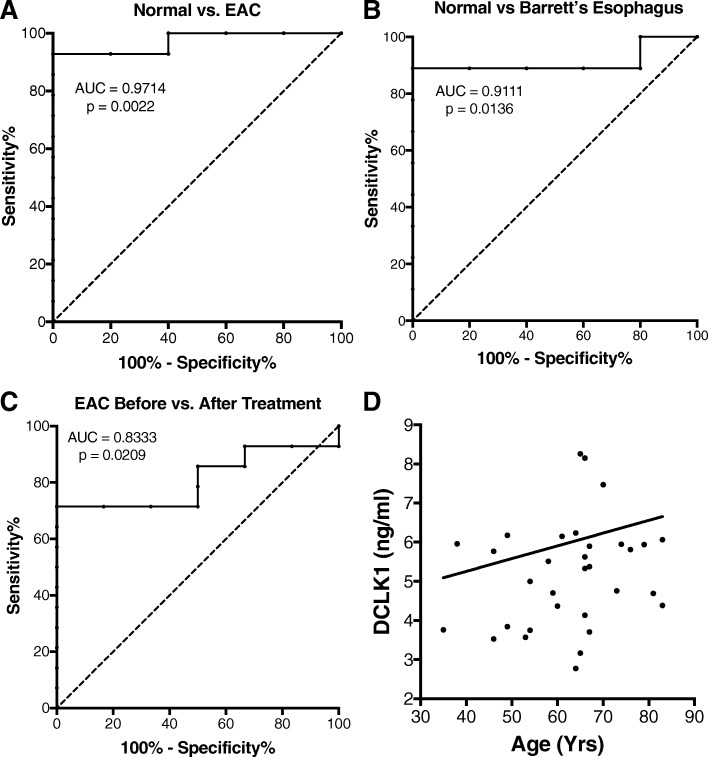
**a**. ROC analysis illustrates that DCLK1 serum levels are a sensitive and specific measure of the presence of esophageal adenocarcinoma (*n* = 14) when compared to controls (*n* = 5) as indicated by an area under the curve (AUC) of 0.9714 (*p* = 0.0016). **b**. Similar results were calculated for Barrett’s esophagus (*n* = 9) compared to controls (AUC: 0.9111, *p* = 0.0136). **c**. Comparison of EAC serum after treatment (*n* = 6) demonstrated a more moderate but significant AUC of 0.8333 (*p* = 0.0209). **d**. Serum levels of DCLK1 are not significantly related to patient age (*p* = 0.66, Pearson r = 0.08)

Analysis of serum DCLK1 levels between patient groups using the Kruskal-Wallis test showed statistically significant differences (*p* = 0.0028). Serum DCLK1 levels were increased by 67% (95% CI: 45.0–89.7%) in patients with EAC prior to treatment (*n* = 14) compared to controls (*n* = 5, Fig. 1). DCLK1 levels in EAC were reduced > 50% following therapy (*n* = 6, Fig. 1). To determine the sensitivity and specificity of serum DCLK1, we compared levels in Barrett’s Esophagus or EAC patients before treatment to normal controls (Figs. 2a and b) and we compared EAC patients before and after treatment (Fig. 2c). In all cases, serum DCLK1 was sensitive and specific with an area under the curve (AUC) of approximately 0.833–0.971 (*p* < 0.021). Together these findings suggest the possibility of using serum DCLK1 as a diagnostic marker for EAC, particularly following successful treatment of early disease. Since controls samples were obtained from patients of significantly younger age (median age = 49 yrs), we assessed DCLK1 serum levels compared to age and found no significant correlation in the total dataset (Pearson r = 0.08, *p* = 0.66; Fig. 2d) or among individual patient subgroups. Comparison of DCLK1 serum levels based on gender also revealed no significant differences (data not shown).

## Discussion

Inflammation is emerging as one of the key hallmarks of cancer [10], and its role is nowhere more apparent than in the gastrointestinal (GI) tract which must maintain homeostasis in the presence of pathogens. Doublecortin-like kinase 1 (DCLK1) is a marker of sensory/secretory tuft cells in the GI tract which have essential roles in the inflammatory response and tumorigenesis. Recent studies in colon and pancreas models of inflammation demonstrate that DCLK1+ cells patrol for injury and initiate the inflammatory response to helminth infection, viral infection, colitis, and pancreatitis. Moreover, in the presence of relevant mutations, DCLK1+ cells become the cell of origin for inflammation-associated pancreatic and colorectal cancers [11]. Like these cancers, EAC is often associated with inflammatory injury and may emerge from regions of intestinal metaplasia. Therefore, investigating of the role of DCLK1 and DCLK1+ cells in EAC and EAC-associated conditions is warranted.

The incidence of EAC has been increasing in the past few decades while the prognosis remains poor. To date, there are no screening markers for EAC detection. This is the first study to demonstrate that serum DCLK1 correlates with disease regression following treatment of EAC. DCLK1 levels were statistically different between EAC patients before and after treatment. Importantly, these findings demonstrate the potential utility of serum DCLK1 as a non-invasive marker of disease regression post-therapy and a potential target for therapeutic intervention. Finally, it is tempting to speculate that observed changes result from the eradication of tumor cells secreting DCLK1, but further investigations are necessary to test this hypothesis.

The significant difference between DCLK1 levels in patients with EAC versus normal controls supports our previous findings [12]. This study was able to show a significant difference between patients who had eradication of their EAC and those with existing EAC, suggesting that DCLK1 may be a viable adjunct biomarker for treatment effect. Further studies will be necessary to assess whether this could also be helpful in identifying patients who have a recurrence after therapy.

This study had several limitations. The patient sample size was small, and length of enrollment did not allow for more than 1 post-treatment DCLK1 measurement. Not all of the patients who had EAC at the time of enrollment were able to provide end of treatment serum samples during this study period. Further evaluation of larger numbers of patients before and after therapy for reduction of serum DCLK1 is required. Although DCLK1 is not unique to EAC, the exclusion criteria for this study eliminated the risk of falsely elevated levels due to other malignancies (e.g. colon, pancreas). However, it will be important for future studies to evaluate whether anti-inflammatory medications are able to impact DCLK1 serum levels as this could be a potential confounding factor. Patients with normal upper endoscopy findings (source of control serum) were younger and more likely to be female compared to other patient subgroups, but DCLK1 levels were not correlated with these factors in this study. Larger scale studies will require the collection of more concordant samples to overcome potential confounding factors. Overall, the findings in this brief report suggest that serum DCLK1 should be assessed for its potential as a non-invasive marker to assess EAC therapy response. Additionally, it may have promise in screening for both initial presentation and disease recurrence.

## Abbreviations

BE: Barrett’s esophagus;
DBE: BE with dysplasia
DCLK1: Doublecortin-like kinase 1
EAC: Esophageal adenocarcinoma
EMR: Endoscopic mucosal resection
EoT: End of treatment
GERD: Gastroesophageal reflux disease
HGD: High grade dysplasia
RFA: Radiofrequency ablation
